# Genomic investigation of a household SARS-CoV-2 disease cluster in Arizona involving a cat, dog, and pet owner

**DOI:** 10.1016/j.onehlt.2021.100333

**Published:** 2021-09-29

**Authors:** Hayley D. Yaglom, Gavriella Hecht, Andrew Goedderz, Daniel Jasso-Selles, Jennifer L. Ely, Irene Ruberto, Jolene R. Bowers, David M. Engelthaler, Heather Venkat

**Affiliations:** a Translational Genomics Research Institute, Pathogen and Microbiome Institute, 3051 W. Shamrell Blvd Ste. 106, Flagstaff, AZ 86005, USA; b Arizona Department of Health Services, Office of Infectious Disease Services, 150 North 18th Avenue, Suite 140, Phoenix, AZ 85007, United States of America; c Centers for Disease Control and Prevention, Center for Preparedness and Response, Career Epidemiology Field Officer Program, 1600 Clifton Rd, Atlanta, GA 30333, USA

**Keywords:** SARS-CoV-2, Companion animals, Pets, Genomic sequencing, One health, RT-PCR, Real-time Polymerase Chain Reaction

## Abstract

Arizona's COVID-19 and Pets Program is a prospective surveillance study being conducted to characterize how SARS-CoV-2 impacts companion animals living in households with SARS-CoV-2-positive individuals. Among the enrolled pets, we identified a SARS-CoV-2-infected cat and dog from the same household; both animals were asymptomatic but had close contact with the symptomatic and SARS-CoV-2-positive owner. Whole genome sequencing of animal and owner specimens revealed identical viral genomes of the B.1.575 lineage, suggesting zoonotic transmission of SARS-CoV-2 from human to at least one pet. This is the first report of the B.1.575 lineage in companion animals. Genetically linking SARS-CoV-2 between people and animals, and tracking changes in SARS-CoV-2 genomes is essential to detect any cross-species SARS-CoV-2 transmission that may lead to more transmissible or severe variants that can affect humans. Surveillance studies, including genomic analyses of owner and pet specimens, are needed to further our understanding of how SARS-CoV-2 impacts companion animals.

## Highlights

• Two companion animals from the same household tested positive for SARS-CoV-2.
• Sequencing of the cat, dog, and pet owner samples revealed identical viral genomes.
• Phylogenetic analysis shows that the viral genomes fall into a locally circulating clade of B.1.575.
• This is the first known report of the B.1.575 lineage in companion animals.
• Evidence of viral transmission from a pet owner to at least one pet in the same household was identified through genomic sequencing.

## 1 Introduction

Natural infection with SARS-CoV-2 in dogs and cats is widely documented [[1], [2], [3], [4], [5]]. Clinical manifestations in companion animals range from asymptomatic infections to severe illness, including respiratory illness [5,6]. As of September 1, 2021, more than 180 dogs and cats have tested positive for SARS-CoV-2 in the United States (US) [7]. Eleven cats, 7 dogs, and one ferret in Arizona have tested positive for SARS-CoV-2 or had evidence of SARS-CoV-2 specific antibodies (internal data).

There is no evidence that companion animals play a role in spreading SARS-CoV-2 to humans. However, transmission from infected people to animals, especially during close contact, has been reported [6,8]. While several states [6,9,10] are conducting surveillance in pets and applying genomic technologies to understand the zoonotic nature of SARS-CoV-2, limited studies have compared viral genome data of pets with owners. This report describes one household cluster of SARS-CoV-2 infection involving a human, a cat and a dog, and provides evidence for recent transmission from the pet owner, identified through whole genome sequencing.

## 2 Arizona's pet program and case report

Arizona's COVID-19 and Pets Program is an ongoing cross-sectional study being conducted to characterize how SARS-CoV-2 impacts companion animals living in households with SARS-CoV-2-positive individuals. Authorized Arizona Department of Health Services (ADHS) project staff identified positive COVID-19 cases using Arizona's Infectious Disease Surveillance System (MEDSIS) and downloaded a line-list for all confirmed COVID-19 cases that tested PCR positive (and had 2+ symptoms) within the past 14 days. Households in Maricopa and Coconino counties in Arizona were contacted by phone for study recruitment within one week of symptom onset, asked if they owned pets, and if yes, were offered to have more information about the project sent to them. Persons interested in the study were emailed informational materials and offered enrollment for their pet(s). If they responded back that they were interested in participating, a home visit was scheduled. After written consent was obtained, trained veterinary and public health staff visited enrolled households to collect blood, nasal, and fecal specimens from pets and complete a questionnaire about pet interactions with owner(s). Eligibility criteria included companion animals (dogs, cats, and ferrets) living in a household with a person who had tested positive for COVID-19 in the past 14 days. Pets must be housed mainly indoors, be up-to-date on their rabies vaccination, and tolerate handling and restraint necessary for routine veterinary care.

This project was deemed an epidemiological investigation that did not involve human subjects' research. Approval was granted by the Translational Genomics Research Institute's (TGen) Animal Care and Use Committee (#20163) and the ADHS Human Subjects Review Board (#20–0017). Through this program, as of September 21, 2021 specimens were collected from 19 households with 56 pets; 15 pets (from 7 unique households) tested positive for SARS-CoV-2. Here we describe one confirmed cluster.

In March 2021, we enrolled a 1-year-old spayed female domestic long-hair cat and 6-year-old neutered male mixed-breed dog. Both were up-to-date on their vaccines and received annual veterinary exams, lived exclusively indoors (with the exception of leashed walks for the dog), and were reported by the owner to show no signs consistent with SARS-CoV-2 infection in animals (e.g. lethargy, coughing, discharge, diarrhea) both at the time of sampling and after testing was completed. Nasal and rectal swabs and a blood specimen were collected from both pets on March 16, 10 days after the pet owner's symptom onset.

The pet owner was a previously healthy, unvaccinated 28-year-old male who resided with his wife (age unknown) and daughter (2 years old). He developed symptoms on March 6 (headache, runny nose, and fatigue), and over the course of illness experienced difficulty breathing, muscle aches, brain fog, and chest pain/tightness. The pet owner was not hospitalized and fully recovered from his illness. Reverse transcriptase polymerase chain reaction (RT-PCR) testing of nasal swabs collected at four consecutive timepoints (March 6, 9, 16, and 25) from the pet owner were positive for SARS-CoV-2.

The pet owner's wife remained asymptomatic and never tested positive for SARS-CoV-2. His daughter tested positive by RT-PCR on March 15, but remained asymptomatic. No travel history was reported for the owner, wife, or daughter, but the owner had close contact with family friends (2 adults and 1 child) via an indoor social gathering hosted outside the family residence within the 14 days prior to illness onset (March 3). These two adult friends developed COVID-19-like symptoms shortly after the gathering and tested positive for SARS-CoV-2 via nasal swabs by antigen testing and RT-PCR.

The owner was the main caretaker of both pets, and reported having close contact with them while symptomatic, including petting/cuddling and providing routine care (e.g., cleaning the litterbox, walking the dog). The dog and cat frequently laid on the couch with the owner, sat on his lap, and slept in the same bed. During the household visit, it was observed that the cat and dog remained in close contact with each other.

## 3 SARS-CoV-2 animal testing and genomic analyses

Collected animal specimens were tested for SARS-CoV-2 by RT-PCR; cycle threshold [Ct] values were recorded for all RT-PCR-positive specimens. The GenScript SARS-CoV-2 Surrogate Virus Neutralization Test (sVNT) Kit was used on all serum specimens, which detects and measures circulating neutralizing antibodies against the SARS-CoV-2 virus. The kit has been widely used to support COVID-19 seroprevalence investigations and is species-interdependent [10].

SARS-CoV-2 was detected in the cat's rectal swab (Ct value = 28) and the dog's nasal swab (Ct value = 35). The cat's nasal swab and dog's rectal swab tested negative. Virus shedding patterns may vary in some animals, including dogs and cats, based on previous studies, which might explain why not all of the swabs tested positive [11]. The cat's serum also had evidence of neutralizing antibodies. The specimen collected from the pet owner on March 16 was available for whole genome sequencing, but specimens from his daughter and two adult friends were unavailable for further analysis.

Whole genome sequencing of specimens obtained from the cat, dog, and pet owner using previously documented methods [12] produced viral genomes with 95%, 84%, and 97% breadth of coverage of the Wuhan reference genome, respectively. The Pango-lineage for all three genomes was identified as B.1.575, which has been reported in >30 countries, with the majority of genomes (90%) originating in the US [13]. This lineage is characterized by a number of amino acid changes that distinguish it from other lineages; all were present in the genomes from the cat, dog, and owner (Table 1).

**Table 1:** Amino acid changes identified in the B.1.575 viral genome sequences obtained from a cat, dog, and pet owner living in the same household, and two related SARS-CoV-2 positive human specimens in Arizona. The bolded mutations^a^ – L18F and Q613H – are unique to these five genomes.

Sample Name	SARS- CoV-2 Protein Names				
	ORF1a	ORF1b	S	N	M
EPI ISL 1525089 (Cat) TGen-CoV-AZ (Dog) EPI ISL 1464779 (Owner) EPI ISL 1525972 EPI ISL 1363573	Q57H, T492I, T85I, T181I	P47S, P323L	**L18F**, S494P, **Q613H**, D614G, T716I, P681H	T205I	I82T

a The L18F and Q613H mutations have been found in approximately 1% of all genomes of the B.1.575 lineage. The L18F mutation has been associated with several variants of concern and may play a role in viral transmission and immune evasion [18]. The Q613H mutation occurs in the ACE-2 receptor binding domain of the spike protein.

**Fig. 1:**
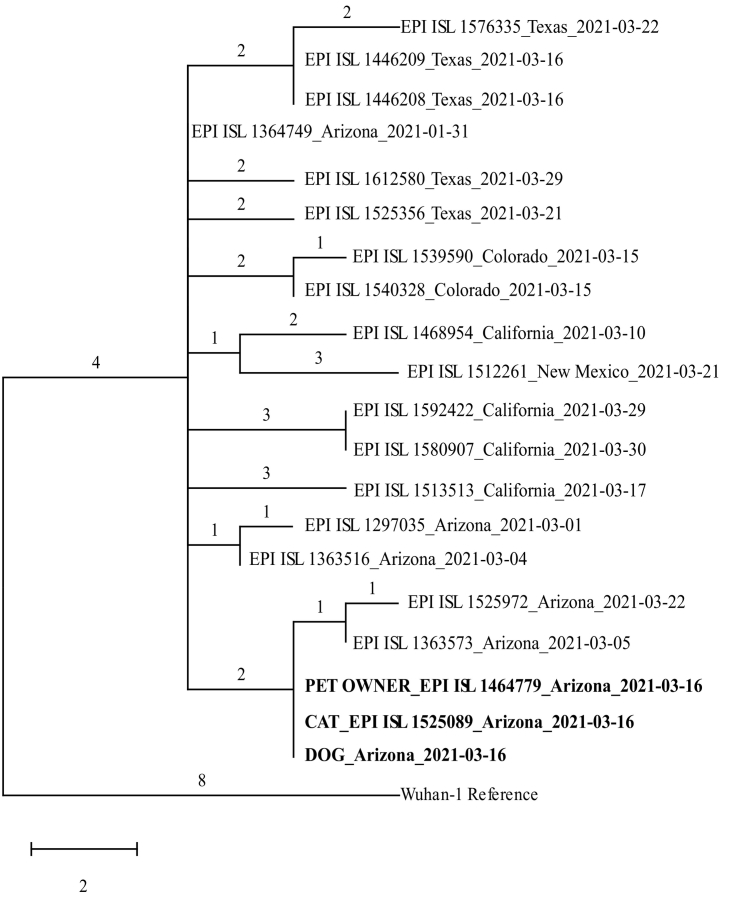
Maximum parsimony phylogenetic tree (CI:0.92, QBC: 77.7%) showing the relationship between the cat, dog, and pet owner viral genomes of the B.1.575 lineage with a subset of 17 SARS-CoV-2 viral genomes from human cases in Arizona and surrounding states collected over a similar timeframe for context using the Wuhan-1 genome as a reference (EPI_ISL_402125). Genomes for owner and cat samples have been published in GISAID (EPI_ISL_1,464,779 and EPI_ISL_1,525,089).

The phylogenetic tree (Fig. 1) shows that the cat, dog, and pet owner viral genomes are identical, and fall into a monophyletic clade with two other Arizona human specimens (with 1 and 2 derived mutations) suggesting they are part of a locally circulating subclade of B.1.575. Two mutations (L18F and Q613H) distinguish the genomes of the two other Arizona specimens from the three cluster specimens. Public health officials investigated potential exposures of the reported human COVID-19 cases from which the genomically-related specimens were collected, but were unable to identify an epidemiological link because these cases were lost to follow-up.

Publicly available genomes from 16 companion animals in the US fall into eight different SARS-CoV-2 lineages, including B.1.1 (*n* = 1), B.1.1.7 (*n* = 5), B.1.2 (*n* = 4), B.1. 234 (n = 1), B.1.429 (n = 1), B.1.526 (*n* = 2), B.1.571 (n = 1), B.1.577 (n = 1). (GISAID Accession IDs: EPI_ISL_ EPI_ISL_1218882–884, 1,241,386, 1,315,074–075, 2,930,556–561, 699,506–509). Among these companion-animal genomes, we identified the following amino acid changes in common with the genomes from the cat, dog, and pet owner specimens: T85I and Q57H in the ORF1a gene; P323L in the ORF1b gene; and D614G in the S gene. This finding is not remarkable, since the majority of SARS-CoV-2 genomes carry these mutations.

## 4 Discussion

The detection of SARS-CoV-2 in a cat and dog from the same household as their symptomatic COVID-19 positive owner and identical viral genomes suggest cross-species transmission, with suspected directionality from human to animal for at least one pet (either the cat or the dog). Given the pets' close contact, we cannot rule out the possibility that one pet infected the other after initially being infected by their owner. We could not conduct longitudinal testing of the cat and dog.

The B.1.575 lineage has not yet been reported in companion animals. Based on available genomic data, there is no evidence of one dominant SARS-CoV-2 lineage causing illness in pets. While genomic comparisons of SARS-CoV-2-infected pets and their owners have been reported internationally [14,15], this may be the first phylogenetic analysis of this nature in the US. Genetically linking SARS-CoV-2 between people and animals, and tracking changes in SARS-CoV-2 genomes of both humans and animals over time, could have broader implications for COVID-19 control and prevention strategies if cross-species SARS-CoV-2 transmission may lead to more transmissible or severe variants that can affect humans. This possibility emphasizes the importance of a collaborative One Health approach [16,17] during animal-related SARS-CoV-2 investigations and when considering new recommendations on how SARS-CoV-2 positive humans should interact with animals.

Our findings reinforce the current Centers for Disease Control and Prevention recommendations [16] that SARS-CoV-2-positive people should avoid or limit close contact with their pets to prevent transmission to—and possible infection of—companion animal species. Humans can transmit SARS-CoV-2 to minks, and mink-to-human transmission has been identified; the possibility of other animals being able to transmit SARS-CoV-2 to humans should be monitored [19]. Additional surveillance studies that include genomic analyses, are needed to further improve our understanding of how SARS-CoV-2 impacts companion animals; these investigations could possibly help identify the spread of SARS-CoV-2 to other animals or people if this issue does emerge.

## Declarations of interest

None.

## Disclaimers

The findings and conclusions in this report are those of the authors and do not necessarily represent the official position of the Centers for Disease Control and Prevention. 

Use of trade names and commercial sources is for identification only and does not imply endorsement by the Centers for Disease Control and Prevention, the Public Health Service, or the U.S. Department of Health and Human Services.

## Funding support

This study/report was supported in part by an appointment to the Applied Epidemiology Fellowship Program administered by the 10.13039/100005663Council of State and Territorial Epidemiologists (CSTE) and funded by the 10.13039/100000030Centers for Disease Control and Prevention (CDC) Cooperative Agreement Number 1NU38OT000297-03-00.

## Authorship statement

None.
